# CRP (C Reactive Protein) level after total knee replacement in Indian population--- does it follow Anglo-Saxon trend?

**DOI:** 10.1186/s42836-020-00043-7

**Published:** 2020-08-27

**Authors:** Sanjay Bhalchandra Londhe, Ravi Vinod Shah, Amit Pankaj Doshi, Shubhankar Sanjay Londhe, Kavita Subhedar

**Affiliations:** 1 Criticare Hospital, Plot No 516, Besides SBI, Telli Galli, Andheri East, Mumbai, Maharashtra 400069 India; 2 Hinduja Healthcare, Mumbai, India; 3 Meril Life Sciences, Vapi, India; 4 MIT college of Engineering, Pune, India; 5 Criticare Hospital, Mumbai, India

## Abstract

**Background:**

This study was to determine how C-reactive protein (CRP) responds after total knee replacement (TKR), including both unilateral and simultaneous bilateral TKR in Indian population and if it follows Anglo-Saxon trend. Published literature from North America and Europe shows CRP value peaks on the 2nd post-operative day and drops to normal by 6–8 weeks. We started the study with null hypothesis.

**Material and methods:**

This is a prospective study, with 50 patients (all females, 25 received unilateral operations and 25 bilateral ones) included. CRP levels were measured, on the 2nd day, 8, 12 and 16 weeks after operation.

**Results:**

In both groups, CRP level rose the 2nd post-operative day. The rise in CRP level was significantly higher in the simultaneous bilateral TKR group than in the unilateral TKR group. In unilateral cases, CRP on the 2nd postoperative day ranged from 65 to 110 mg/l with average level of 80 mg/ml. In bilateral TKR cases, CRP level on the 2nd postoperative day was between 110 and 180 mg/l with a mean of 140 mg/ml. The CRP level returned to normal in about 40% of unilateral TKR patients 8 weeks after operation, while in 92% (23 out of 25) of bilateral simultaneous TKR patients it stayed at a high level 8 weeks post-op and did not come back to normal. At 12 weeks CRP decreased to normal in all 100% of unilateral TKR patients and 32% of bilateral TKR patients. At 16 weeks, CRP was normal in all bilateral TKR patients.

**Conclusion:**

60% of our unilateral TKR patients and 92% of our simultaneous bilateral TKR patients did not achieve a normal CRP 8 weeks after operation. These findings are significant as CRP is commonly used as a very sensitive indicator of postoperative joint infection. Hence we conclude that in the Indian TKR patients the CRP values take longer time to return to normal than in their Anglo-Saxon counterparts. Published results regarding the normal levels of CRP in unilateral TKR should not be extrapolated to simultaneous bilateral TKR patients.

## Background

This study was to determine the response of CRP after TKR surgery, including both unilateral and simultaneous bilateral TKR. According to the previously published literature from North America and Europe, CRP value peaks on the 1st and 2nd postoperative day and it gradually comes down to normal by 6–8 weeks post-operatively [1–4]. The CRP was chosen as a marker as it has better sensitivity and specificity than the ESR for the diagnosis of prosthetic joint infection [5–10]. According to Shih *et al* [6], ESR sensitivity is 82% and specificity is 85% while CRP sensitivity is 86% and specificity is 92%. Early diagnosis of prosthetic joint infection is extremely important as it helps to start treatment earlier.

## Aim

To determine the trend of CRP in Indian patients undergoing TKR, including both unilateral and simultaneous bilateral TKR, and to see whether it follows the trend identified in North American and European population and to determine whether there is a difference in the CRP pattern in unilateral *versus* simultaneous bilateral TKR patients.

## Materials & methods

Fifty patients undergoing TKR between January 2016 and May 2016 were included in this study. Sample size was estimated to be 20 in each group (unilateral and simultaneous bilateral TKR) with alpha error being 0.05, beta error 0.05 and power 0.95. Considering drop-outs, the number was rounded to 25. Twenty five patients had unilateral TKR and simultaneous bilateral TKR. All 50 patients were female and the mean age in the unilateral group was 67 years and it was 69 years in the simultaneous bilateral TKR group. Local ethics committee approval and informed consent from the patients were obtained. The CRP was turbidimetrically measured, by drawing 3 ml of blood, on a computer controlled automated robotic auto-analyzer (Vitros − 250, Thermo Fisher Scientific). The normal CRP value was < 10 mg/l. CRP levels were measured preoperatively, on the 2nd day, 8, 12 and 16 weeks after operation. All the patients had a normal CRP before the operation. Inclusion criteria included: patients with advanced osteoarthritis of the knee (Grade 4, bone on bone) affecting the quality of life and daily activities. Patients who had been preoperatively diagnosed as having inflammatory arthritis, such as rheumatoid arthritis, systemic lupus erythematoses and postoperative adverse events like skin site infection, deep prosthetic joint infection, and urinary tract infection were excluded from this study. There were no differences in basic parameters between the groups in terms of age, sex, BMI, American Society of Anesthesiologists grade, preoperative CRP and white blood cells count, severity of osteoarthritis and operative time (Table 1). All the patients were operated on by a single surgeon and assistants. TKR was performed in a standard fashion with a tourniquet. Pre- and postoperatively, both groups were given standard prophylactic antibiotic, i.e., one dose 2 h before operation and two more doses 6 and 12 h after operation. All patients received a posterior stabilized knee implant (Maxx Freedom Knee). Before implant placement, all patients in both groups received a periarticular cocktail injection using a standard 7-zone technique. At the end of TKR procedure, all the patients received adductor canal block. Postoperative pain management involved multi-modal analgesia approach in the form of intravenous injection of paracetamol, followed by oral paracetamol, intravenous injection of tramadol, followed by oral tramadol, and buprenorphine transdermal patch. Non steroidal anti-inflammatory drugs (NSAIDS) were administered and they were given only as a rescue analgesic. Pregabalin (75 mg) was started on the day before the operation and was continued for 2 months after the operation.

**Table 1:** Comparison of patient characteristics between Unilateral and Bilateral TKR group

Parameters	Unilateral TKR Group	Bilateral TKR Group	***p*** value
**Number of patients (n)**	*n* = 25	*n* = 25	**–**
**Mean Age (yrs)**	67.5 ± 6.01 (58–82)	69.1 ± 4.57 (60–79)	0.2946
**Mean BMI (kg/m**^**2**^**)**	28.9 ± 7.1	29.5 ± 6.6	0.7583
**Gender**			
Males	*n* = 0 (0%)	*n* = 0 (0%)	
Females	*n* = 25 (100%)	*n* = 25 (100%)	
**ASA Grade**			
Grade 1	*n* = 6	*n* = 5	0.7354
Grade 2	*n* = 14	*n* = 15	0.7767
Grade 3	*n* = 5	*n* = 5	1.000
**Mean Preoperative CRP**	4.5 ± 1.6	4.7 ± 1.2	0.6194
**Mean Preoperative WBC Count**	8550 ± 1280	8220 ± 1150	0.3008
**Preoperative clinical diagnosis**			
OA (Grade 4, bone on bone)	*n* = 25 (100%)	*n* = 25 (100%)	
RA	*n* = 0 (0%)	*n* = 0 (0%)	
**Mean operative time (minutes)**	78.5 ± 16.5	82.6 ± 14.9	0.3595
**NSAIDS consumption (mg)**	190 ± 38.19	194 ± 36.29	0.7059

## Results

**Fig. 1:**
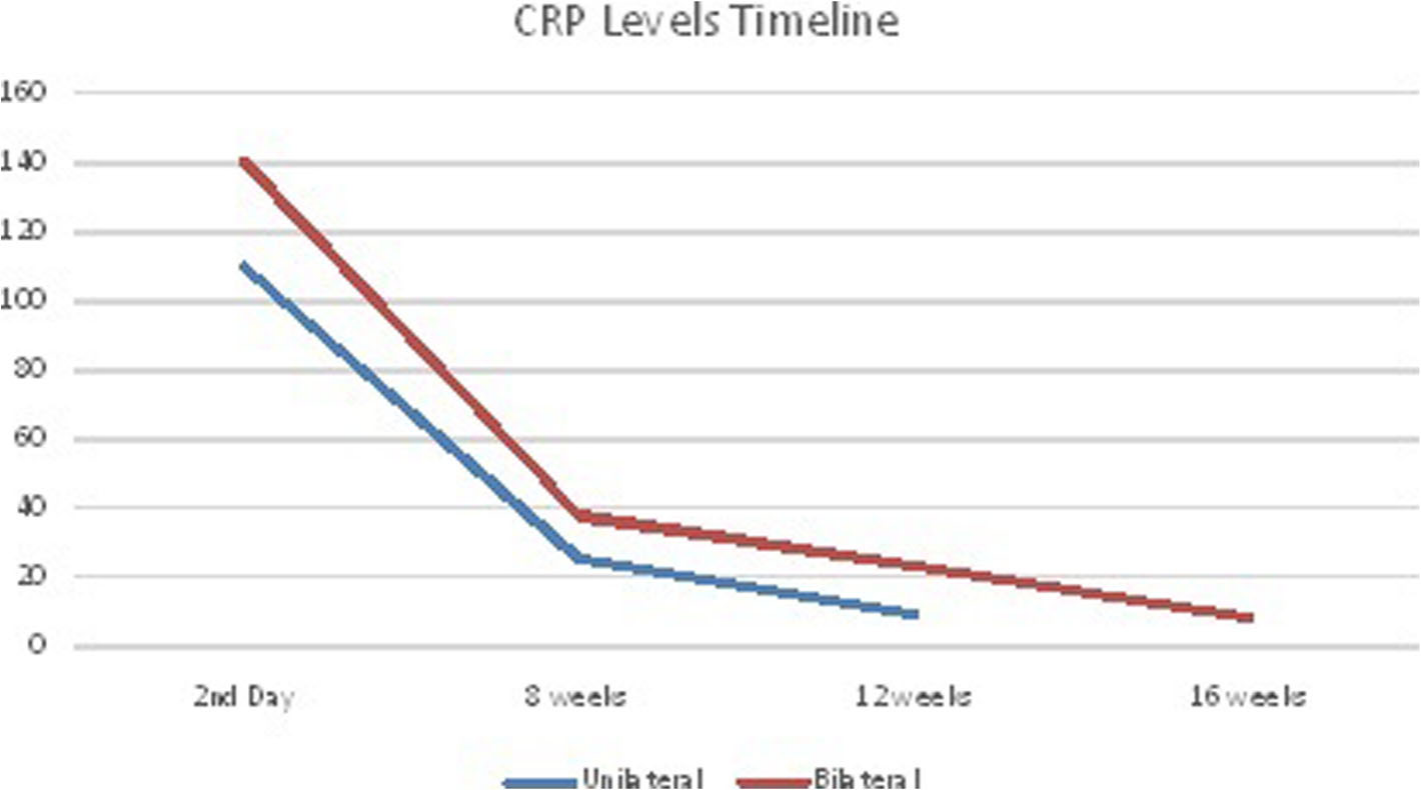
CRP Levels Timeline for Unilateral and Bilateral TKR

All the patients had normal CRP before the operation. In both the groups, CRP level shot up on the 2nd post-operative day. The rise in CRP level was significantly higher in the simultaneous bilateral TKR group than in the unilateral TKR group. In unilateral cases, CRP on the 2nd postoperative day ranged from 65 to 110 mg/l with a mean of 80 mg/l. In bilateral TKR cases, CRP on the 2nd postoperative day was 110–180 mg/l, with a mean of 140 mg/l (Table 2). The CRP level returned to normal in about 40% of unilateral TKR patients 8 weeks after operation, while in 92% (23 out of 25) of bilateral simultaneous TKR patients, it was still elevated 8 weeks after operation and did not drop to normal. Twelve weeks after operation, CRP decreased to normal in all 100% of unilateral TKR patients and in 32% of bilateral TKR patients. At 16 weeks, CRP was normal in all bilateral TKR patients (Tables 3 and 4 and Fig. 1). All the patients were followed up for 1 year. There were no postoperative complications in any of the patients. The operating time in the unilateral group was 78.5 ± 16.5 min and that in the simultaneous bilateral group was 82.6 ± 14.9 min (*p* value = 0.3595). There was no difference in NSAIDS consumption between the unilateral (190 ± 38.1881 mg) and bilateral TKR group (194 ± 36.2859 mg), with *p* value being 0.7059. In the unilateral TKR group, CRP was normal in 40% of the patients at 8 weeks and in 100% of the patients at 12 weeks. On the other hand, in bilateral TKR group, CRP was normal in 8% of the patients at 8 weeks and in 32% of the patients at 12 weeks and in 100% patients at 16 weeks.

**Table 2:** Observations made on the 2nd postoperative day

	CRP Levels (mg/dl)		
TKR type	Minimum Value	Maximum Value	Mean Value
Unilateral (*n* = 25)	65	110	80
Bilateral (*n* = 25)	110	180	140

**Table 3:** % of the patients whose CRP became normal after various timelines

TKR Type	Normal% at 8 weeks	Normal% at 12 weeks	Normal% At 16 weeks
Unilateral	40%	100%	–
Bilateral	8%	32%	100%

**Table 4:** CRP levels (mg/l) at various timelines

	Unilateral Knee			Bilateral Knee		
Post Op Timeline	Minimum	Maximum	Mean	Minimum	Maximum	Mean
2nd Day	65	110	80	110	180	140
At 8 weeks	9	25	19	9.5	40	37.5
At 12 weeks	5	9.5	7	8.5	30	23.5
At 16 weeks	–	–	–	6.5	9	8

## Discussion

C-reactive protein (CRP) is an annular (ring-shaped), pentameric protein found in blood plasma, whose circulating concentrations rise in response to inflammation. It is an acute-phase protein of hepatic origin that increases following interleukin-6 secretion by macrophages and T cells. The macrophages are present in the bone and bone marrow and less commonly exist in the skeletal muscle. The bone and bone marrow injury happening during TKR can cause elevation of CRP [2]. TKR is more traumatic than total hip replacement and more likely to induce CRP elevation. Various North-American and European studies have shown that the CRP level increases significantly on the 2nd postoperative day and it decreases from a peak on the 2nd postoperative day, returning to normal value 6 to 8 weeks after operation [1–4]. Dunsmuir *et al* [1] showed that the peak level of the CRP occurred on the 2nd and 3rd postoperative days after TKR and total hip replacement. Although the peak appeared at the same time, the level after TKR was, on average, 50% greater than that after THR (ANOVA, *p* = 0.002). Larsson *et al* [2] measured the level of C-reactive protein (CRP) and erythrocyte sedimentation rate (ESR) by serial measurements after four types of uncomplicated elective orthopedic surgeries. The surgeries included total hip arthroplasty (primary, *n* = 109; and revisions caused by aseptic loosening, *n* = 9), unicondylar knee arthroplasty (*n* = 39), and lumbar microdiscectomy (*n* = 36). In all patients, CRP levels increased after surgery, reaching a peak on the second day after knee arthroplasties (140 ± 46 mg/l). C-reactive protein levels usually dropped to normal (less than 10 mg/l) within 21 days after surgery. They did not find any correlation between CRP response and the type of anesthesia, amount of bleeding, transfusion, operation time, administered drugs, age or gender. Erythrocyte sedimentation rate peaked about 5 days after surgery, followed by a slow and irregular decrease. ESR often remained elevated 42 days after operation. They concluded that the CRP level is a better diagnostic marker for the early detection of postoperative infections than ESR. They postulated that the rapid decline in CRP after uncomplicated orthopedic surgery will be interrupted by a second rise or by a persisting elevation if there is an infectious complication in the postoperative period. Choudhary et al [4] studied the changes in ESR and C-reactive protein to establish normal values after total hip or knee arthroplasty. They found that the ESR decreased from 1.68 (± 0.017) to 1.57 (± 0.014) on the first postoperative day and thereafter rose to 1.60 (± 0.019), 1.75 (± 0.015), and 1.74 (± 0.011) on the third, seventh and fourteenth days, respectively. The ESR returned to preoperative levels 6 to 8 weeks after operation. The C-reactive protein level increased significantly on the first postoperative day and then decreased from a peak on the second day, attaining nearly normal levels 6 to 8 weeks after operation. They concluded that the CRP is a more sensitive indicator of deep postoperative infection than plasma viscosity. The result of our study is not in agreement with this published literature. Nearly 60% of our unilateral TKR patients and 92% of all of our simultaneous bilateral TKR patients did not achieve a normal CRP at 8 weeks after operation. These findings are significant as CRP is often used as a very sensitive indicator of postoperative joint infection [5–13]. Majority of patients from both groups did not achieve normal CRP level 8 weeks after surgery. To the best of our knowledge, this is the first study of its kind from Indian subcontinent/Mainland China that analyzed the trend of CRP after TKR. Moreover, this is the first study in the world to show the trend of CRP in patients undergoing simultaneous bilateral TKR. Only in one Iranian study, did Nazem *et al* [14] show that the level of CRP reached its maximum on the second postoperative day and then showed a downward trend up to 1 month after the operation. The study showed that the CRP did not reach its preoperative level during the first postoperative year. In our study, Indian TKR patients took longer time to return to normal CRP level. The unilateral TKR patients took almost 8 to 12 weeks and the bilateral TKR patients took 12 to 16 weeks for the CRP level to become normal. What is more, the simultaneous bilateral TKR patients had statistically significant higher CRP levels than the unilateral TKR patients at various time intervals (on the 2nd post-op day, 8 weeks and 12 weeks after operation), with *p* value < 0.001, as indicated by Two-sample *t* test (Table 5). BK Lee *et al* [15] had shown that CRP elevation in early postoperative period post TKR can be because of periprosthetic joint infection, genitourinary infection, respiratory infection and deep vein thrombosis. This is not the case in our study population as patients with post-operative adverse events like skin site infection, deep prosthetic joint infection, urinary tract infection; respiratory tract infection and deep vein thrombosis were excluded from this study. We hypothesize that Indian patients taking longer time for their CRP value to return to normal than the western population can be due to underlying pro-inflammatory state, excess truncal subcutaneous fat, physical inactivity and protein deficiency. To the best of our knowledge, there were no studies about the pattern of CRP values after simultaneous bilateral TKR patients. As there is a paucity of literature concerning the CRP pattern after TKR in Indian/Asian population, further studies are required at other centers in India and other Asian and South East Asian countries to throw more light on the difference in the CRP response after TKR procedure.

**Table 5:** Two sample t test, *p* value calculation between unilateral and bilateral TKR

Follow up	Unilateraltkr CRP Value	Bilateral TKR CRP value	P value
2nd day, Mean ± SD	80.00 ± 13.48	140.00 ± 19.63	< 0.001
8 weeks, Mean ± SD	19.00 ± 8.48	37.50 ± 07.36	< 0.001
12 weeks, Mean ± SD	07.00 ± 0.96	23.50 ± 11.09	< 0.001

Our study has some limitations. First, both unilateral and simultaneous bilateral TKR group had only female patients. This is not unusual as numerous studies have shown that women are more prone to the development of advanced symptomatic osteoarthritis [16–18]. Second, we excluded the patients who had pre-existing inflammatory conditions like rheumatoid arthritis and systemic lupus erythematoses from the study. These patients usually have a high preoperative CRP. In future, it will be useful to analyze whether CRP level return to pre-operative level by 12–16 weeks after TKR in this group of patients with inflammatory arthritis.

## Conclusion

Hence, we conclude that in Indian TKR patients, the CRP level takes longer time to return to normal. The unilateral TKR patients took almost 8 to 12 weeks for the CRP value to come to normal. The simultaneous bilateral TKR patients took 12 to 16 weeks for the CRP level to become normal. This is of utmost importance in clinical practice as CRP is often used as sensitive marker to diagnose post TKR prosthetic joint infection. Hence the CRP value trend reported in western literature should not be applied while assessing postoperative prosthetic joint infection in Indian patients. Use of standalone CRP level value as an indicator of postoperative joint infection should be used with caution since it is not an absolute value but the relative reading/trend which is of significance in these cases. As there are no studies about CRP response after simultaneous bilateral TKR, the published literature regarding the normal levels of CRP in unilateral TKR should not be extended to simultaneous bilateral TKR group.

## Data Availability

Not Applicable.

## Abbreviations

CRP: C Reactive Protein
TKR: Total Knee Replacement
NSAIDS: Non Steroidal Anti Inflammatory Drugs
